# Gastric adenocarcinoma causing biliary obstruction without ductal dilatation: a case report

**DOI:** 10.1186/s13256-019-1972-4

**Published:** 2019-03-09

**Authors:** Karl Vaz, Raphael P. Luber, Catriona McLean, Jan Frank Gerstenmaier, Stuart K. Roberts

**Affiliations:** 1 Alfred Hospital, 55 Commercial Rd, Melbourne, VIC 3004 Australia; 2 Austin Hospital, 145 Studley Rd, Heidelberg, VIC 3084 Australia

**Keywords:** Gastric adenocarcinoma, Billroth II, Jaundice, Non-dilated biliary tree

## Abstract

**Introduction:**

Gastric adenocarcinoma is a known complication of partial gastrectomy. Jaundice from gastric adenocarcinoma usually occurs in the setting of hepatic nodal or parenchymal metastasis. This case demonstrates an unusual level of biliary obstruction from gastric adenocarcinoma.

**Case presentation:**

An 84-year-old Caucasian man was diagnosed as having a new gastric adenocarcinoma at the level of the gastroenteric anastomosis of a prior Billroth II gastrectomy after presenting with painless jaundice. He had a non-dilated biliary tree on radiographic imaging despite evidence of large bile duct obstruction on liver biopsy. The obstruction was managed with endoscopic wire-guided stenting of the malignant tumor.

**Conclusions:**

The unusual finding of a non-dilated biliary tree in the face of obstructive jaundice is likely to have resulted from the unusual post-surgical anatomy and hence distal level of obstruction. Endoscopic duodenal stenting is a novel method of managing obstructive jaundice in gastric adenocarcinoma.

## Introduction

Gastric adenocarcinoma is a known long-term complication of partial gastrectomy [1]. Jaundice in the setting of gastric adenocarcinoma largely occurs due to intrahepatic parenchymal or hepatic nodal metastases [2–4]. Here we present an unusual case of an 84-year-old man diagnosed as having gastric adenocarcinoma following presentation with obstructive jaundice in the setting of a previous Billroth II gastrectomy for peptic ulcer disease. The lack of biliary ductal dilatation on non-invasive imaging studies in this patient led to diagnostic uncertainty. Prior reports of obstructive jaundice in the face of a non-dilated biliary tree pre-date the advent of magnetic resonance cholangiopancreatography (MRCP) [5, 6], and are unusual with the high sensitivity of this radiological imaging technique today.

## Case presentation

An 84-year-old Caucasian man with a distant past history of Billroth II gastrectomy for peptic ulcer disease and cholecystectomy for cholelithiasis was admitted to hospital for investigation of painless jaundice following referral from his general practitioner (GP).

He had a 1-month history of anorexia, weight loss, malaise, and painless progressive jaundice. An examination revealed normal vital signs, icterus without stigmata of chronic liver disease, and a soft abdomen with no organomegaly. Biochemistry demonstrated: microcytic anemia with hemoglobin of 108 g/L, mean corpuscular volume 72 fL, and ferritin 4557 μg/L; obstructive cholestasis with bilirubin of 164 umol/L, alkaline phosphatase (ALP) 2167 units/L, gamma-glutamyl transferase (GGT) 857 units/L, aspartate transaminase (AST) 225 units/L, and alanine transaminase (ALT) 301 units/L; modestly raised inflammatory markers with white cell count (WCC) of 12.77 × 10^9^/L and C-reactive protein (CRP) 80 mg/L; and hypoalbuminemia (24 g/L) with a normal international normalized ratio (INR) of 1.2 and platelet count of 294 × 10^9^/L. Cancer antigen 19-9 (CA 19-9) was raised at 132 kU/L.

**Fig. 1:**
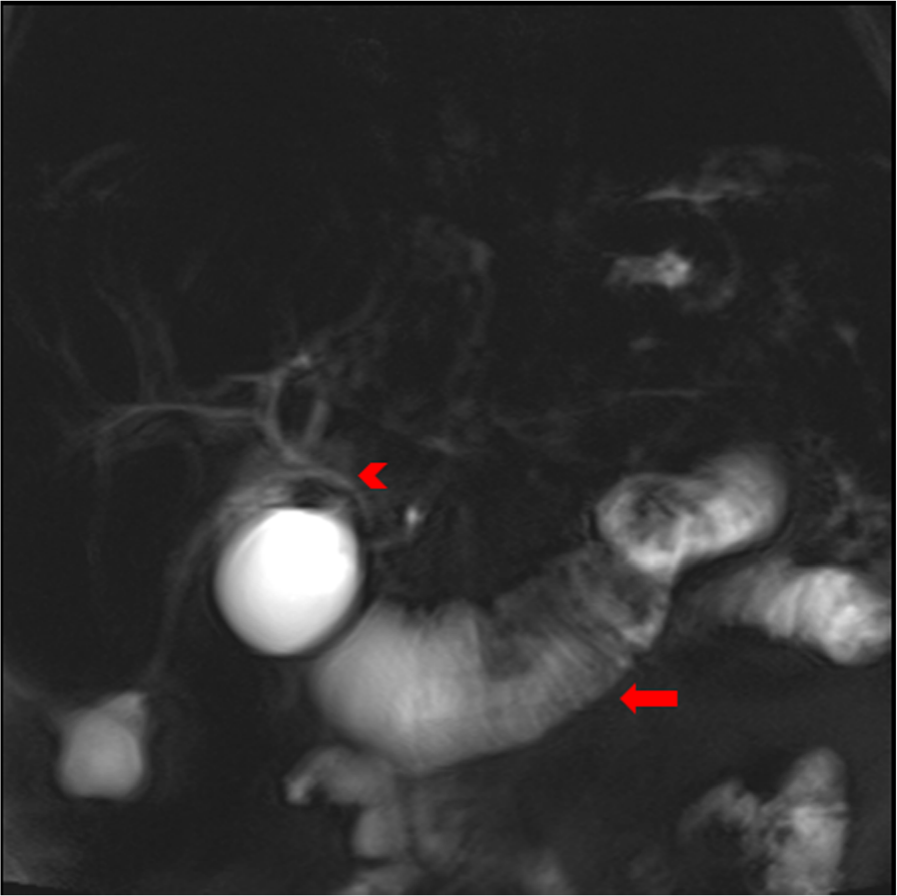
At magnetic resonance cholangiopancreatography, coronal T2-weighted half-Fourier acquisition single-shot turbo spin-echo image shows a markedly dilated afferent duodenal limb (*arrow*) and a non-dilated, normal-appearing biliary tree (*arrowhead*)

His common bile duct, liver parenchyma, pancreas, and portal vein were of normal appearance on abdominal ultrasonography, with a subsequent MRCP showing no dilatation of the biliary tree and no evidence of any intraductal, pancreatic, or hepatic lesions. The MRCP did, however, exhibit dilatation of the afferent duodenal limb being 4.8 cm in maximal width (Fig. 1) and a single enlarged portal hilar lymph node measuring 13 mm in its short axis. Given the discordant lack of biliary dilatation on imaging, a liver biopsy was performed.

**Fig. 2:**
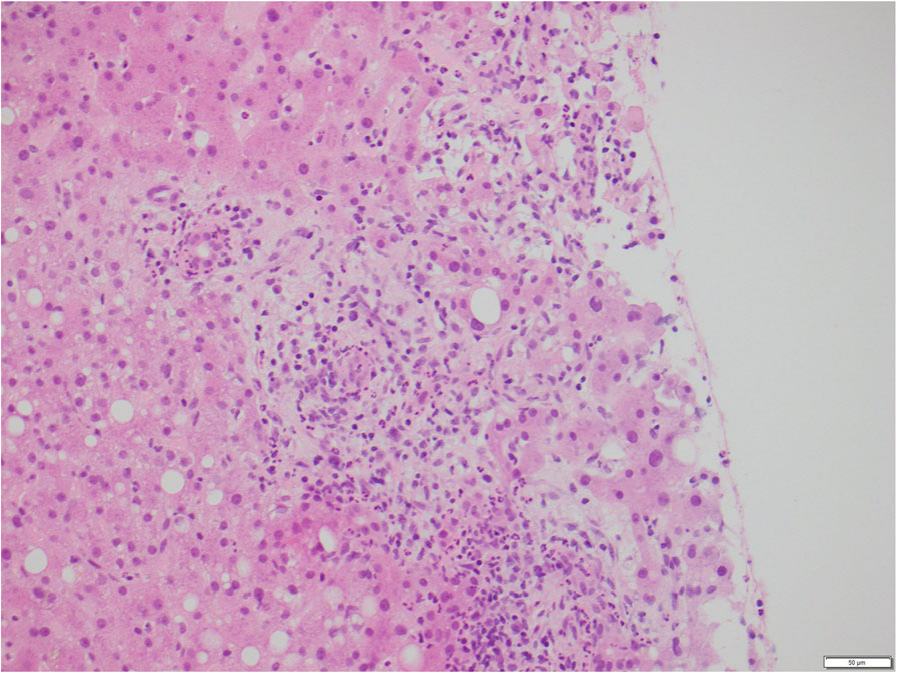
Hepatic biopsy demonstrating edematous portal tract, infiltration of neutrophils, and reactive changes in the bile duct epithelium

**Fig. 3:**
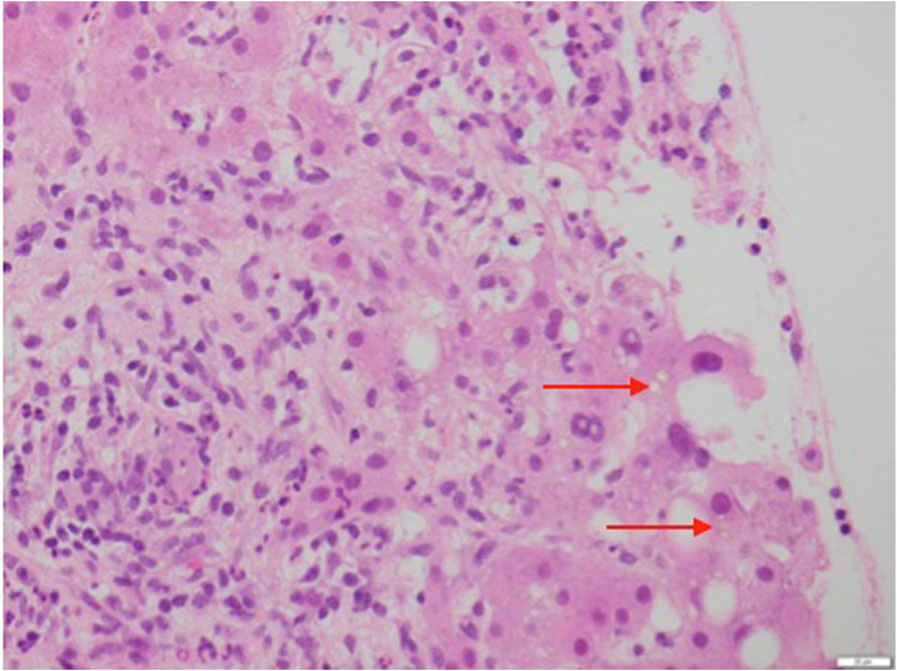
Hematoxylin and eosin × 400 magnification, highlighting intrahepatic bile and edematous tracts with inflammation including eosinophils with ductal proliferation. The top arrow is showing intrahepatic bile, whilst the bottom arrow shows oedematous portal tracts

**Fig. 4:**
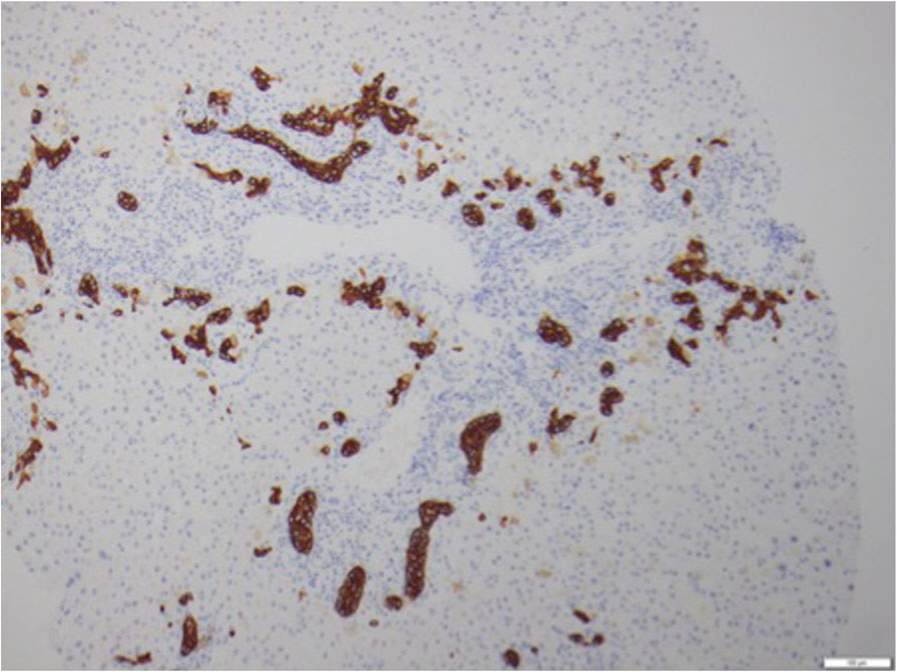
Cytokeratin 7 immunoperoxidase highlighting the ductular proliferation

Histopathology demonstrated edematous portal tracts with inflammation within the portal tracts including neutrophils, which were not seen within the lumen of the ductal epithelial cells (Figs. 2 and 3). Furthermore, lobular bile accumulation was present as was ductular proliferation at the edge of the portal tracts, highlighted by cytokeratin immunoperoxidase (Fig. 4). Focal steatosis was present with no distinct zonal pattern but with a tendency to be periportal. A suspicion of cholangitis was raised and treated with appropriate antibiotics, but with no clinical improvement. The histopathology and clinical picture taken together was felt to be most in keeping with acute large bile duct obstruction. A full hepatic biochemical screen investigating for infective and autoimmune causes was unremarkable.

**Fig. 5:**
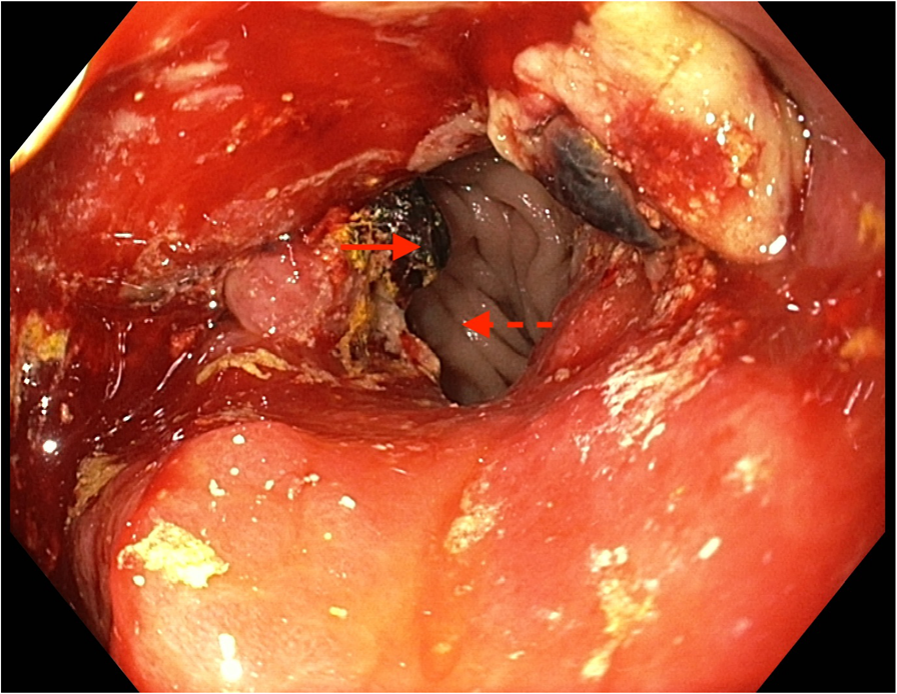
Diagnostic gastroscopy demonstrating ulcerated gastric carcinoma (*solid-line arrow*) at gastroenteric anastomosis with efferent limb in view (*dashed-line-arrow*) and inability to visualize afferent limb secondary to tumor obstruction

A gastroscopy was subsequently performed to investigate the enteric abnormalities noted on MRCP, revealing a markedly deformed remnant stomach with a mass lesion at the gastroenteric anastomosis suspicious for malignancy (Fig. 5). The afferent limb was stenosed and unable to be intubated preventing visualization of the major duodenal papilla. Biopsies of the mass lesion confirmed moderately differentiated gastric adenocarcinoma.

**Fig. 6:**
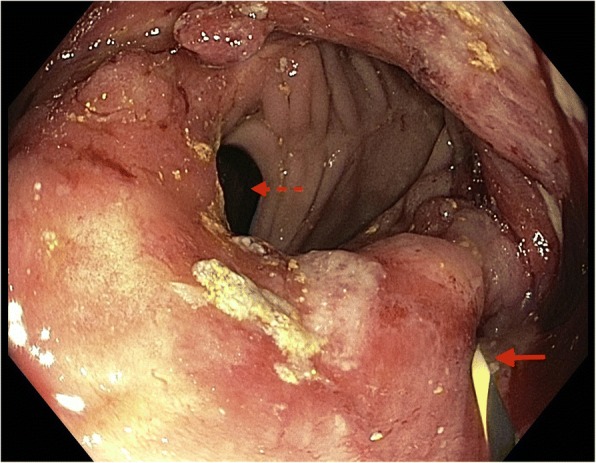
Wire inserted into afferent limb (*solid-line arrow*) with efferent limb on view (*dashed-line arrow*)

The biliary obstruction was managed by endoscopic wire-guided stent insertion through the obstructing tumor into the afferent duodenal limb (Fig. 6) with subsequent resolution of jaundice (bilirubin 16 umol/L) and significant improvement in liver enzymes (ALP 424 units/L, GGT 346 units/L, ALT 33 units/L). As our patient was not a candidate for surgical intervention or chemotherapy due to locoregional disease extent and frailty, he was eventually discharged home with palliative support following a period of in-patient rehabilitation.

## Discussion

This case report presents a case of biliary obstruction without radiographic dilatation, secondary to gastric adenocarcinoma in a patient with a previous Billroth II procedure. Gastric adenocarcinoma is a known but uncommon complication of partial gastrectomy, especially after Billroth II procedure [1]. The gastrojejunal anastomotic site is the most common location of malignancy following Billroth II gastrectomy, although the exact mechanisms as to why this occurs are unclear [1, 7].

Jaundice is a rare complication of gastric malignancy and portends a poor prognosis [8]. The vast majority of cases of jaundice in the setting of newly diagnosed or progression of known gastric cancer result from obstruction of the biliary tree secondary to malignant lymph nodes, most commonly at the hepatoduodenal ligament, or as a result of hepatic metastases leading to intrahepatic obstruction [2–4]. To the best of our knowledge, this is the only described case in the literature in which the primary gastric malignancy has resulted in biliary obstruction rather than nodal disease or metastatic extension of the tumor. This is owing to our patient’s unusual post-surgical anatomy.

The lack of demonstrable upstream biliary dilatation by appropriate cross-sectional radiographic imaging of the abdomen led to diagnostic uncertainty in this case. Endoscopic retrograde cholangiopancreatography (ERCP) is the gold standard to investigate the cause of biliary obstruction. However, its invasive nature carries a small but not insignificant risk of morbidity and mortality particularly after Billroth II gastrectomy. As such, non-invasive radiographic investigations, namely abdominal ultrasonography and MRCP, are widely used as preferential first-line tests in the diagnostic algorithm for obstructive jaundice. The diagnostic performance of MRCP compared with ERCP has been studied at length since its introduction to clinical practice in 1991, with a 2006 systematic review demonstrating a sensitivity of 81–94% and 87–100% for malignancy and dilatation, respectively [9].

MRCP is particularly advantageous in the diagnostic evaluation of patients with post-surgical anatomy, such as Billroth II procedure, as ERCP may not be technically feasible in these patients [10, 11]. The absent finding of radiographic biliary obstruction in this case is unlikely to be a failure of the imaging modality but rather a result of the anatomic level of obstruction due to the post-surgical anatomy. Although the exact mechanism leading to lack of biliary dilatation in this case is not entirely clear, we theorize that the distensibility of the duodenum precluded further upstream dilation of the common bile duct. Treatment of this patient with an endoscopic duodenal stent presents a novel mode of management in such a patient with biliary obstruction.

## Conclusion

This case report highlights an unusual mode of biliary obstruction in the face of gastric adenocarcinoma. Whereas the main cause of jaundice in gastric malignancy is due to hepatic nodal or parenchymal metastasis, the primary tumor distal to the biliary tree resulted in obstruction in this case. This is the first case in the literature to describe such an anatomical location of obstructive cholestasis. Furthermore, failure to detect the biliary obstruction through MRCP is an unusual occurrence in anatomic obstruction of the biliary system. This is most likely due to the distensibility of the afferent limb of the duodenum and serves as a reminder that non-invasive radiographic techniques are imperfect diagnostic modalities in investigating obstructive jaundice.

## Abbreviations

ALP: Alkaline phosphatase
ALT: Alanine transaminase
AST: Aspartate transaminase
CA 19-9: Cancer antigen 19-9
CRP: C-reactive protein
ERCP: Endoscopic retrograde cholangiopancreatography
GGT: Gamma-glutamyl transferase
GP: General practitioner
INR: International normalized ratio
MRCP: Magnetic resonance cholangiopancreatography
WCC: White cell count
